# Early protoplast culture and partial regeneration in *Cannabis sativa:* gene expression dynamics of proliferation and stress response

**DOI:** 10.3389/fpls.2025.1609413

**Published:** 2025-06-06

**Authors:** Daniel Král, Josef Baltazar Šenkyřík, Vladan Ondřej

**Affiliations:** Department of Botany, Faculty of Science, Palacký University Olomouc, Olomouc, Czechia

**Keywords:** protoplast isolation, cell proliferation, gene expression, stress response, microcallus formation, Cannabis culture, oxidative stress, *in vitro*

## Abstract

*Cannabis sativa* L. is a plant of significant interest due to its high content of biologically active compounds, durable fibers, and bioeconomic potential. Despite recent progress in protoplast isolation, complete plant regeneration from cannabis protoplasts remains unachieved, highlighting gaps in protoplast-to-plant systems. This study reports the second successful establishment and partial regeneration of cannabis protoplast cultures, and investigates their molecular dynamics, marking a significant step forward. We demonstrated that the age of donor material is critical for the protoplast isolation, with the optimal source being 1–2-week-old leaves from *in vitro*-grown seedlings. Cultivation in a modified medium developed for *Arabidopsis thaliana* supported initial cell divisions and microcallus formation. Transcriptomic analyses of cell proliferation and stress response markers indicate that the cultured protoplasts were viable, re-entered the cell cycle, and exhibited oxidative and abiotic stress resilience. These findings enhance our understanding of cannabis cell biology and lay the groundwork for a protoplast-based regeneration system, paving the way for advanced applications in biotechnology.

## Graphical Abstract

Workflow for isolation, cultivation, and molecular analysis of cannabis protoplasts. Created in BioRender. Král, D. (2025) https://biorender.com/p09w255.

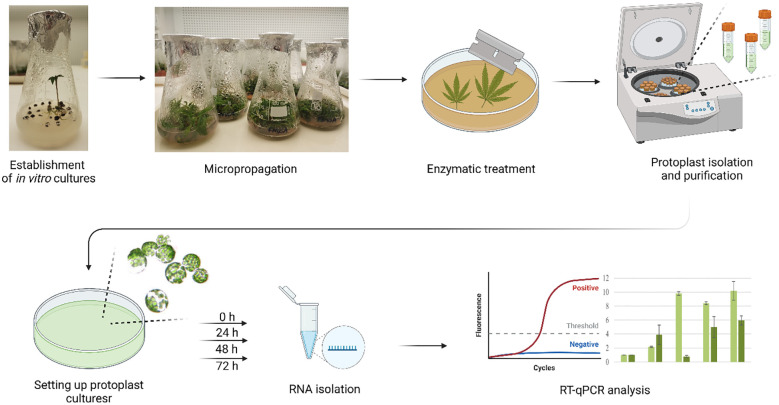

## 1 Introduction

Tissue culture techniques are essential in modern plant biotechnology, enabling controlled *in vitro* cultivation under sterile conditions. Among these, micropropagation stands out as a widely adopted method, facilitating the large-scale cloning of plants (Davey and Anthony, 2010). However, the lack of standardized protocols for *Cannabis sativa* L. micropropagation continues to be a significant limitation (Stephen et al., 2023).

While micropropagation focuses clonal propagation, protoplast-based technologies unlock versatile possibilities by removing the cell wall. These applications include cellular dedifferentiation, genetic transformation, genome editing, and expression system analysis, as well as the production of secondary metabolites (Jiang et al., 2013; Aoyagi, 2011; Hesami et al., 2021). Under optimal conditions, protoplasts can regenerate into whole plants, enabling advanced applications such as cloning, non-chimeric genetic modifications, ploidy modification, and interspecies somatic hybridization (Jiang et al., 2013; Adhikary et al., 2021; Hesami et al., 2021).

In recent years, there has been a notable increase in research activity focused on cannabis protoplasts, particularly in the optimization of isolation methods, their application for transient gene expression, and targeted genome editing. However, despite these advances, data on the *de novo* regeneration of cannabis from protoplasts remain lacking (Hesami et al., 2021).

The complexity of protoplast isolation lies in its reliance on multiple factors, including plant genotype, source material, cultivation, and enzymatic conditions, which require precise species-specific optimization to achieve high yield and viability (Evans and Bravo, 2013). The earliest documented attempt to isolate cannabis protoplasts was made as early as 1979. The study examined the effects of enzymes, solution osmolarity, and source material type, using young and old leaves and calluses for isolation. The highest reported concentration was 10^5^ cells/ml, achieved using pectinase and driselase. While the feasibility of isolation was demonstrated, essential details such as cell viability or the amount of input material were not published (Jones, 1979). It was not until 2007 that cannabis protoplasts were used in a study investigating cell death. This study described the composition of enzymatic solutions (cellulase, macerozyme, and pectolyase), but data on concentration and viability were not provided (Morimoto et al., 2007). Significant progress has been made over the past decade. Flaishman et al. (2019) investigated the isolation of cannabis protoplasts but did not disclose their methodology. Subsequently, Lazič (2020) optimized the isolation process from calluses, young leaves, and hypocotyls of *in vitro* germinated cannabis. The best results were achieved with etiolated hypocotyls, yielding a concentration of 1.8 · 10^5^ cells/ml with 25.7% viability. The year 2021 marked a turning point with two landmark studies. Matchett-Oates et al. (2021) focused on young leaves derived from *in vitro* cultivated apical explants, achieving yields of up to 7.8 · 10^6^ cells/g with 72% viability. A similar approach was applied by Beard et al. (2021) using an enzymatic solution developed for *A. thaliana* enriched with pectolyase, resulting in yields of 2.27 · 10^6^ cells/g and 82% viability. Further advances followed in 2022. Kim et al. (2022) optimized enzymatic treatments for fully developed greenhouse-grown leaves, yielding 9.7 · 10^6^ cells/g, although viability was not reported. Zhu et al. (2022) compared hypocotyl and cotyledon-derived protoplasts, with cotyledons providing the highest recorded yield of 1.15 · 10^7^ cells/g and 98.5% viability. The most recent study, published in 2024, focused on protoplast isolation from calluses derived from hypocotyls. Using a medium containing 2-aminoindan-2-phosphonic acid (AIP), an inhibitor of lignin synthesis, significantly enhanced protoplastization efficiency by weakening the cell wall. The average yield was 8.8 · 10^4^ cells/ml, with a viability rate of 92.1% (Monthony and Jones, 2024). A comprehensive summary of available publications focusing on the isolation of cannabis protoplasts, along with detailed information on the isolation conditions, is provided in **Table 1**.

**Table 1:** Protoplast Isolation studies in cannabis.

Variety or cultivar	Source material	Isolation conditions	Density or yield	Viability [%]	Reference
Afghan	Young and old leaves (grown in a greenhouse) and calluses	1% pectinase; 1% driselase; 10% sorbitol; 1% MgCl_2_; 1% KH_2_PO_4_; 4–16 h	10^5^ cells·ml^-^¹	-	Jones (1979)
Mexican	Leaf cells	1% cellulase; 0.2% macerozyme; 0.4M mannitol; 0.1% pectolyase; 88mM sucrose; 30 °C; 4 h; gentle stirring	-	-	Morimoto et al. (2007)
Finola	Calluses, young leaves, hypocotyls from *in vitro* seedlings	1.5% cellulase; 0.4% macerozyme (leaves); 1% cellulase; 0.1% macerozyme (hypocotyls); 3–14 h; 25 °C; gentle stirring	1.8·10^5^ cells·ml^-^¹	25,7	Lazič (2020)
Cannbio-2; THC/CBD rich	Young leaves from *in vitro* apical explants	2.5% cellulase; 0.3% macerozyme; 0.7M mannitol; 20mM MES; 10mM CaCl_2_; 20mM KCl; 16 h; 28 °C; no stirring	7.8·10^6^ cells·g^-^¹	72,0	Matchett-Oates et al. (2021)
Cherry x Otto II: Sweetened and others	Young leaves from *in vitro* apical explants	1.25% cellulase; 0.3% macerozyme; 0.4M mannitol; 0.075% pectolyase; 20mM MES; 0.1% BSA; 10mM CaCl_2_; 20mM KCl; 16 h; room temperature; gentle stirring	2.27·10^6^ cells·g^-^¹	82,0	Beard et al. (2021)
Abacus	Fully developed mature leaves (grown in a greenhouse)	1.5% cellulase; 0.4% macerozyme; 1% pectolyase; 0.4M mannitol; 0.5M MES; 0.1% BSA; 8mM CaCl_2_; 15 h; 23 °C; gentle stirring	9.7·10^6^ cells·g^-^¹	-	Kim et al. (2022)
3 high-THC; 2 high-CBD; 3 intermediate	Hypocotyls and cotyledon leaves	2.5% cellulase; 0.5% macerozyme; 0.4M mannitol; 0.03% 2-mercaptoethanol; 20mM MES; 0.1% BSA; 10mM CaCl_2_; 20mM KCl; 6 h; 22 °C; gentle stirring	1.15·10^7^ cells·g^-^¹	98,5	Zhu et al. (2022)
Finola	Calluses	1.25% cellulase; 0.3% macerozyme; 0.4M mannitol; 0.075% pectolyase; 20mM MES; 0.1% BSA; 10mM CaCl_2_; 20mM KCl; 16 h; 25 °C; gentle stirring	8.8·10^4^ cells·g^-^¹	92,1	Monthony and Jones (2024)

*De novo* regeneration, a crucial attribute of protoplasts, enables transformative applications in genetic engineering and cultivar development. In cannabis, however, this regenerative potential remains largely unexplored, creating a critical bottleneck for advancements in protoplast-based biotechnologies (Li et al., 2022; Hesami et al., 2021; Monthony and Jones, 2024).

To date, there are only two published reports on the regeneration of cannabis protoplasts. The first, by Flaishman et al. (2019), found that just 4% of protoplasts survived for 48 hours in liquid culture, with no plant regeneration achieved. More recently, Monthony and Jones (2024) provided the most comprehensive results to date, describing partial regeneration during the initial cultivation period. Cell division was observed after six days, and microcallus formation occurred after three weeks. However, cell viability declined significantly at this stage, preventing further development. The study also highlighted the critical role of culture density: no response was observed at 0,5 · 10^5^ cells/mL, limited division occurred at 1 · 10^5^ cells/mL, and the best results were achieved at 2 · 10^5^ cells/mL.

The initial study of cannabis protoplast isolation also involved interspecific somatic hybridization with tomato. However, no hybrid plant regeneration was achieved (Jones, 1979). Significant progress towards transient transformation techniques for cannabis protoplasts was initially reported by Matchett-Oates et al. (2021) and Beard et al. (2021). Both studies employed plasmid DNA carrying reporter expression cassettes, achieving transformation efficiencies of 23% and 31%, respectively. The following year, Kim et al. (2022) reported a transformation efficiency of 55.3% by using fusion genes involved in cannabinoid biosynthesis tagged with GFP, which allowed the visualization and localization of these enzymes in subcellular compartments. Later that year, Zhu et al. (2022) demonstrated a transformation efficiency of 75.4% using GFP to track the nuclear localization of the transcription factor CsMYC2. Transient transformation of protoplasts has emerged as a powerful tool for evaluating gRNA constructs for CRISPR/Cas9-based genome editing. Zhang et al. (2021) developed a system to validate gRNA specificity, enabling efficient mutagenesis of target gene regions. The phytoene desaturase marker gene was used as the editing target. This work culminated in the creation of the first stable genetically modified cannabis plant, albeit not derived from protoplasts.

Protoplastization enhances chromatin accessibility, initiating stochastic gene expression changes that drive dedifferentiation and foster regenerative potential. During this process, cells shift from their original somatic programming to a new state, enabling division and developmental plasticity (Zhao et al., 2001; Xu et al., 2021). However, successful dedifferentiation depends on external phytohormones, particularly auxins (Aux) and cytokinins, without which cells rapidly degenerate. Aux alone promote redifferentiation, while their combined action induces chromatin decondensation and re-entry into the cell cycle (Vissenberg et al., 2000; Zhao et al., 2001). As a marker of the activated Aux signaling pathway, the *IAA-2* gene can be used (Abel et al., 1994, 1995; Yang et al., 2017; Gao et al., 2018; Král et al., 2022).

Proliferating cell nuclear antigen (PCNA) is a highly conserved protein found in all eukaryotes and archaea, playing a central role in DNA replication and various other nuclear processes. Its primary function is to coordinate the recruitment of replication-associated proteins, ensuring efficient replication progression. Additionally, PCNA is involved in translesion DNA synthesis, base and nucleotide excision repair, mismatch repair, recombination, chromatin remodeling, sister chromatid cohesion, and cell cycle regulation (Lee et al., 2019). For this reason, the *PCNA* gene is a widely used cell division marker and has been applied in protoplast cultures of various species, including tobacco and cucumber (Williams et al., 2003; Cápal and Ondřej, 2014), and was therefore selected for use in the present study.

Abscisic acid (ABA) is a pivotal phytohormone that orchestrates various physiological processes in plants. It plays a central role in managing responses to abiotic stresses and regulating developmental transitions from embryogenesis to senescence (Finkelstein et al., 2008). ABA regulates the expression of key gene families including protein phosphatases 2C (*PP2C*) and late embryogenesis abundant (*LEA*) genes. LEA proteins are critical protectants, safeguarding cells against dehydration and damage caused by extreme temperatures and salinity (Liu et al., 2019). In contrast, PP2C proteins function as negative regulators of ABA signaling, maintaining a balanced and appropriate adaptive response to environmental stresses (Park et al., 2009). To evaluate the extent of abiotic stress experienced by the cells in our system, we analyzed the expression of representative members of both LEA and PP2C gene families.

Protoplast viability is significantly affected by oxidative stress, caused by the accumulation of reactive oxygen species (ROS). While ROS are natural byproducts of cellular metabolism, their excessive generation during cell wall enzymatic digestion can disrupt the plasma membrane and exert toxic effects (Cassells and Curry, 2001; Zhang et al., 2022). Plants counteract ROS through various antioxidant mechanisms such as the ascorbate-glutathione cycle and catalases, which convert hydrogen peroxide into water via enzymatic activity of ascorbate peroxidases (APX) or catalases (CAT) (Caverzan et al., 2012; Ratanasanobon and Seaton, 2013). The success of protoplast cultivation depends on the timely activation of antioxidant systems, which typically peak within the first three days. This early cultivation phase is critical in determining whether protoplasts will successfully progress into the cell cycle (Kapur et al., 1993; Siminis et al., 1994; Ondřej et al., 2010; Moricová et al., 2013; Cápal and Ondřej, 2014). To assess the activation of antioxidant responses, we monitored transcript levels of *CAT* and *APX* genes by RT-qPCR throughout the cultivation period.

This study addresses key challenges associated with cannabis protoplast isolation and regeneration. By investigating expression changes of selected genes, we provide valuable insights into the physiological state and regenerative potential of cannabis protoplast cultures. Our findings help to establish a foundation for protoplast-to-plant regeneration methods in cannabis.

## 2 Materials and methods

### 2.1 Plant material

Industrial hemp varieties with varying cannabidiol (CBD) content, including *Cannabis sativa* L. ‘USO 31’, ‘Fédora 17’, ‘Finola’, ‘Futura 75’, ‘Fibror 79’, ‘Santhica 27’, and ‘Santhica 70’, were cultivated in a greenhouse using a standard peat-perlite mixture (seeds provided by Agritec Plant Research Ltd, Šumperk). Mature plants served as sources for protoplast isolation and explants for *in vitro* culture initiation. Additionally, *in vitro* cultures derived from cultivar ‘Eletta Campana’ and from CBD-rich cannabis strain ‘Tangerine Dream’ were included in the study. Both were obtained from the collection of the Department of Botany, Faculty of Science, Palacký University, Olomouc.

### 2.2 Establishment of *in vitro* cultures

**Figure 1:**
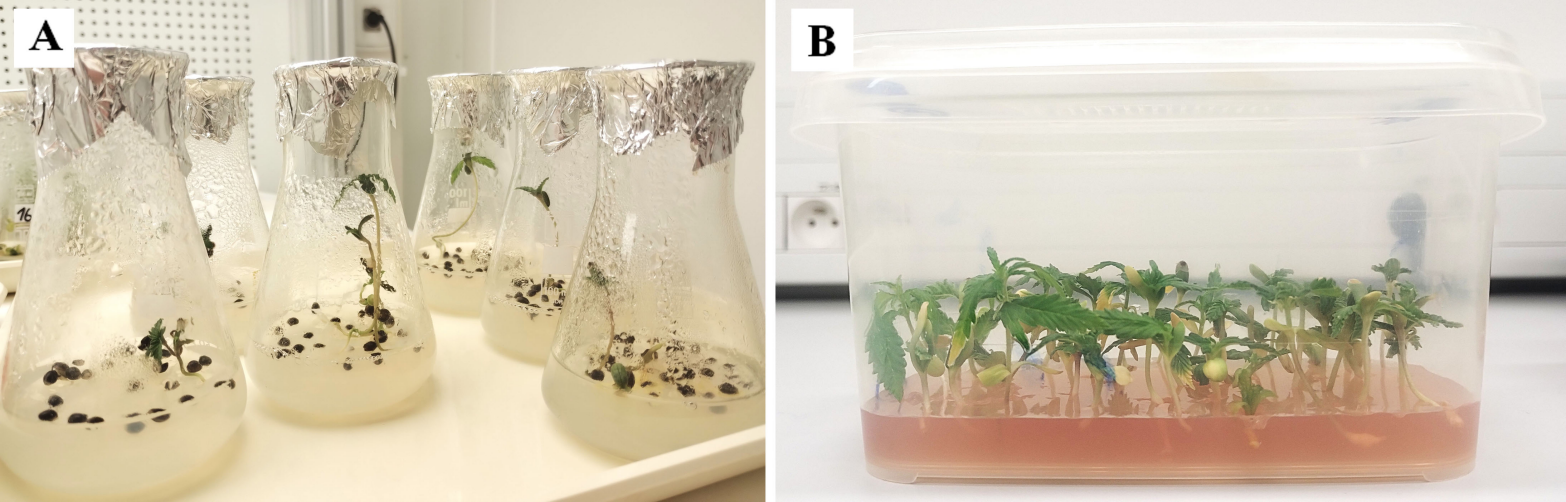
Photographs of *in vitro* germinated seedlings of *C*. *sativa* ‘USO 31’ **(A)** one-week-old. **(B)** two-weeks-old.

The surface sterilization of seeds was initiated with a 2-minute treatment in 96% ethanol (with agitation), followed by rinsing with autoclaved sterile distilled water. Subsequently, seeds were treated with a 20% commercial bleach solution (SAVO, Unilever, Czech Republic) containing a surfactant for 20 minutes (with shaking), then rinsed three times with sterile distilled water. Sterilized seeds were transferred to culture vessels containing ½ MS medium and germinated in a growth chamber for up to one month (**Figure 1**). Newly germinated plants were transferred weekly to culture media (KM1–5) and subjected to micropropagation for six months, subculturing at two-week intervals.

Nodal segments from different plant parts underwent a 20-minute rinse in distilled water, followed by surface sterilization with 70% ethanol (20 seconds, shaking) and a 20% bleach solution with a surfactant (20 minutes, shaking). Explants were maintained in culture media (KM1–5) and subcultured biweekly.

Standard growth chamber conditions were maintained at 22°C, 40% relative humidity, and a 16-hour light/8-hour dark photoperiod. To identify optimal growth conditions, various media formulations and phytohormone combinations were tested (**Table 2**). Additionally, the efficacy of different gas exchange systems was evaluated, including sealed environments with aluminum foil and breathable systems using surgical tape (Ševčíková et al., 2016 – modified).

**Figure 2:**
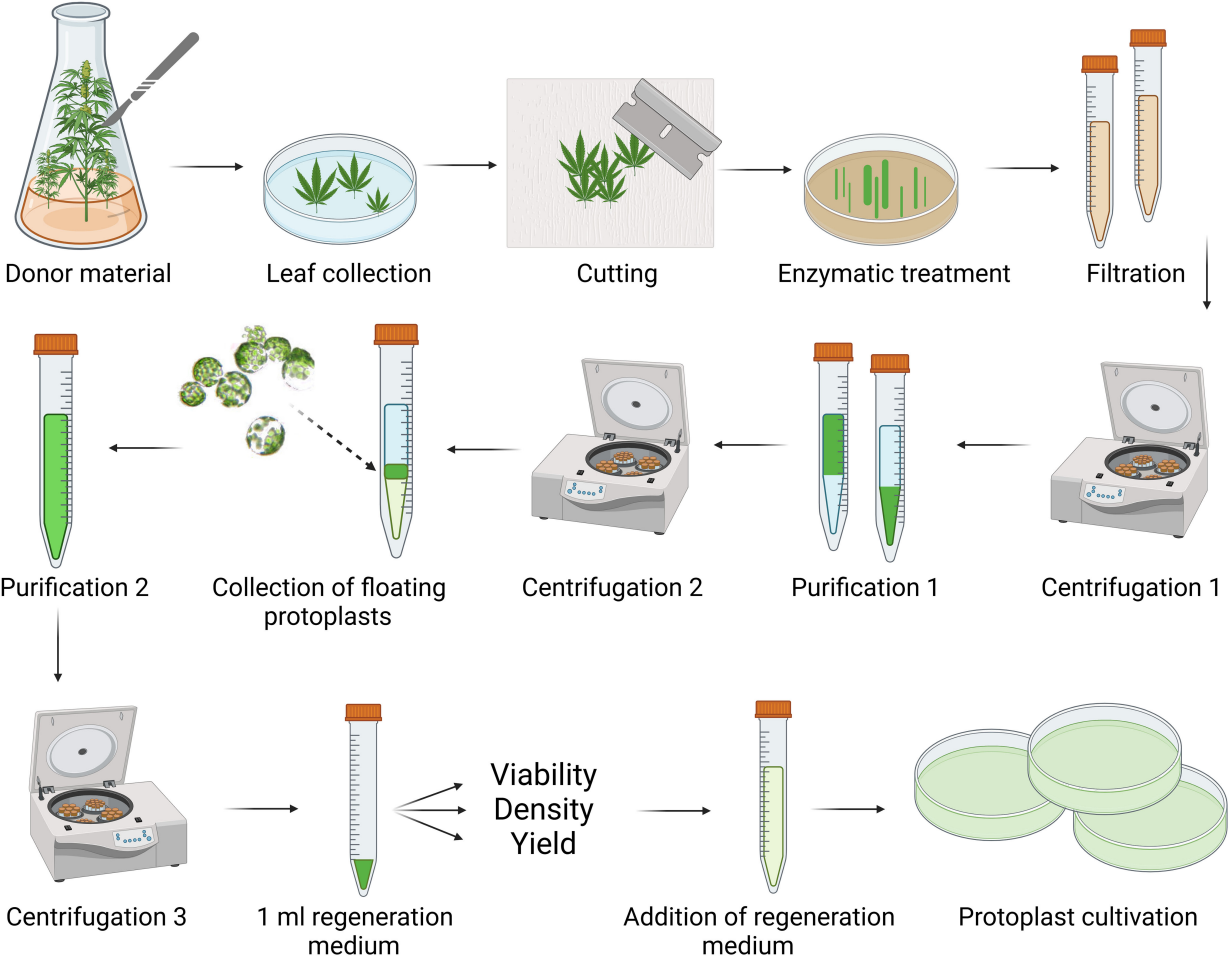
Schematic workflow for cannabis protoplast isolation and purification. Created in BioRender. Král, D. (2025) https://BioRender.com/g42t222.

**Table 2:** Composition of culture media used for *C. sativa* micropropagation.

Culture media (1 l)	Medium [g]	Growth regulators [mg]
KM1	ViVi 6 (6.17)	TDZ (0.3)
KM2	ViVi 6 (6.17)	mT (0.1); IBA (0.1)
KM3	DKW (5.58)	mT (0.1); IBA (0.1)
KM4	ViVi 6 (6.17)	2iP (0.1)
KM5	DKW (5.58)	2iP (0.1)

+ 7 g of agar; 22 g of sucrose; 20 mg of ascorbic acid; pH 5.8; 1.2 ml of PPM; 133 mg of ampicillin and 66 mg of chloramphenicol.

### 2.3 Solutions for the protoplast isolation

Five enzyme solutions (ER1a–d; ER2) were tested to optimize protoplast isolation efficiency. The ER1a solution was directly adopted from Matchett-Oates et al. (2021), while ER1b–d were modified versions based on protocols from Matchett-Oates et al. (2021); Beard et al. (2021), and Kim et al. (2022). The ER2 solution was adopted from Beard et al. (2021). To prepare the enzyme solutions, all components were dissolved in 80 ml of distilled water preheated to 55°C under continuous stirring (**Table 3**). The volume was then adjusted to 100 ml, the pH was set to 5.8, and the solutions were sterilized by filtration to preserve enzymatic activity.

**Table 3:** Composition of enzymatic and washing solutions for the cannabis protoplast isolation.

Components	ER1a	ER1b	ER1c	ER1d	ER2	W5	W1	W2
Cellulase	2.5%	2.5%	2.5%	2.5%	1.25%	–	–	–
Macerozyme	0.3%	0.3%	0.3%	0.3%	0.4%	–	–	–
Pectolyase	–	0.075%	1%	–	0.075%	–	–	–
Mannitol	0.7 M	0.7 M	0.7 M	–	0.4 M	–	0.7 M	0.4 M
Glucose	–	–	–	5 mM	–	5 mM	–	–
MES	20 mM	20 mM	20 mM	10 mM	20 mM	10 mM	20 mM	20 mM
KCl	20 mM	20 mM	20 mM	5 mM	20 mM	5 mM	20 mM	20 mM
CaCl_2_	10 mM	10 mM	10 mM	125 mM	10 mM	125 mM	10 mM	10 mM
NaCl	–	–	–	154 mM	–	154 mM	–	–
BSA	–	–	–	–	0.1%	–	–	0.1%

Three washing solutions (W5, W1, and W2) were evaluated to enhance protoplast viability and yield. The W5 solution was prepared following the protocol of Matchett-Oates et al. (2021), while W1 was adapted and modified from the same study. The W2 solution was prepared according to Beard et al. (2021). All components were dissolved in 300 ml of distilled water to prepare the solutions under continuous stirring (**Table 3**). The volume was then adjusted to 500 ml, the pH was set to 5.8, and the solutions were sterilized by filtration to ensure sterility without compromising component stability.

### 2.4 Protoplast isolation

Protoplasts were isolated from the leaves of *C. sativa* plants at different developmental stages and cultivation conditions, including *in vitro*-germinated seedlings, *in vitro*-grown plants, and *ex vitro*-cultivated plants. The schematic workflow for protoplast isolation and purification is illustrated in **Figure 2**. Centrifugation parameters were optimized within the 700–1200 rpm and 5–12 minutes to maximize yield and viability.

Leaves were aseptically immersed in sterile distilled water. After removing the stem and apical sections, the leaves were quickly sliced into strips (0.5–1 mm wide) and transferred to Petri dishes containing 6 ml of enzyme solution. Five enzyme formulations were tested to identify optimal conditions for enzymolysis, which was conducted at 25°C in the dark without shaking for 2–16 hours. Digestion was terminated by adding a washing solution, with three formulations evaluated for their effectiveness. The protoplast suspension was filtered through a 72-µm nylon mesh, and residual plant material was rinsed with washing solution. The filtrate was transferred to a 10-ml glass centrifuge tube, and subjected to centrifugation using a discontinuous sucrose density gradient. Two protocols were compared: (1) resuspension of the sediment in 4 ml of washing solution followed by an overlay of 2 ml of 20% sucrose, or (2) resuspension of the sediment in 4 ml of 20% sucrose and overlaying with 2 ml washing solution. Protoplasts floating at the interface were collected, adjusted with the washing solution, and centrifuged again before resuspending in 1 ml of regeneration medium. Viability was determined using fluorescein diacetate (FDA) vital staining. Protoplast concentration and overall yield were quantified using a Bürker counting chamber.

### 2.5 Protoplast culture

The regeneration medium (RM) was prepared by dissolving 1.1 g of MS basal medium and 77.02 g of sucrose in 400 ml of distilled water (dH__2__O). The solution was supplemented with 1 mg of indole-3-acetic acid (IAA), 0.25 mg of 2,4-dichlorophenoxyacetic acid (2,4-D), and 0.25 mg of benzylaminopurine (BAP). The volume was adjusted to 500 ml with dH__2__O, and the pH was set to 5.8. The medium was sterilized by filtration through a 0.22 µm membrane filter (Mathur *et* Koncz, 1998 – modified).

Protoplasts exhibiting viability levels above 60% or below 15% were adjusted to a final concentration of 10^6^ cells/ml using the requisite volume of RM medium. The prepared suspension was distributed into 2-ml culture vessels and incubated in darkness at 25 °C. Samples were collected at 24-hour intervals over 3 days for RT-qPCR analysis to assess gene expression dynamics under these conditions.

### 2.6 Two-step RT-qPCR

Total RNA was extracted using the Spectrum Plant Total RNA Kit (Sigma-Aldrich, Prague, Czech Republic). To remove residual genomic DNA, the RNA samples were treated with DNase I Amplification Grade (Sigma-Aldrich, Prague, Czech Republic) and assessed for quality and DNA contamination via agarose gel electrophoresis. RNA concentration and purity were measured based on the A260/A280 ratio using a NanoDrop 2000 spectrophotometer (ThermoScientific, Prague, Czech Republic). Complementary DNA (cDNA) was synthesized with a SensiFAST™ cDNA Synthesis Kit (Bioline, Prague, Czech Republic). PCR reaction with end-point analysis was performed to verify the correct design of primers and their specificity. The second step of RT-qPCR was conducted using the SensiFAST SYBR No-ROX Kit (Bioline, Prague, Czech Republic) on a CFX Connect Real-Time PCR Detection System (Bio-Rad Laboratories, Inc., Hercules, California, U.S.). Data analysis was conducted using the CFX Maestro™ software, with melting curve dissociation analysis employed to confirm the specificity of PCR products. Relative gene expression levels were calculated using the Pfaffl method (Pfaffl, 2001) and normalized against a control sample – young leaves of *in vitro*-grown seedlings of the ‘USO 31’ cultivar. Normalization was performed relative to freshly isolated protoplasts for genes not expressed in leaves. Following established recommendations (Deguchi et al., 2021), the reference gene EF-1 encoding elongation factor 1, was used for normalization. A detailed list of the analyzed genes and the primer sequences employed is presented in **Supplementary Table S1** (**Supplementary Material**).

### 2.7 Statistical evaluation

The relative expression data were statistically analyzed using SPSS Statistics software. A one-way analysis of variance (ANOVA) was conducted, followed by two *post-hoc* tests: the Dunnett test for assessing significant differences relative to the control (Leaf) and Tukey’s Honestly Significant Difference (HSD) test for evaluating differences among protoplast cultivation samples (**Supplementary Table S2**). Significant differences are indicated in the graphs by asterisks for the Dunnett test (p ≤ 0.01 “**”; p ≤ 0.05 “*”) and circles for the Tukey’s HSD test (p ≤ 0.01 “○○”; p ≤ 0.05 “○”). Graphs display relative expression values as means ± standard deviation, calculated from four technical and two to three biological replicates.

## 3 Results

### 3.1 Influence of the cultivation conditions

The success of protoplast isolation may depend on the cultivation conditions of donor material. Although no direct improvements in protoplast yield were observed in this study, visual differences in growth and overall vitality were noted. KM5 emerged as the most universally effective medium. In contrast, KM1 induced the development of a dwarf phenotype, progressing to fasciation and extensive vitrification (**Supplementary Figure S1**). Media KM2–4 did not cause major abnormalities and were more suitable than KM5 for certain varieties. Additionally, DKW and ViVi6 media supported healthy cannabis growth without notable visual differences.

Initially, explants derived from *ex vitro* nodal segments exhibited restricted growth, with newly formed leaves frequently vitrified. These effects diminished with continued cultivation. Nevertheless, long-term cultivation led to reduced growth, a decline in regeneration capacity, and increased hyperhydricity in most cultivars. These adverse outcomes were particularly pronounced when aluminum foil tightly sealed culture vessels. Conversely, transitioning to breathable closures using surgical tape significantly reduced or delayed these issues, suggesting that improved gas exchange plays a pivotal role in maintaining culture health.

### 3.2 Optimization of protoplastization

An enzymolysis duration of 16 hours provided the best results and was adopted as the standard. Shorter incubation times (2, 4, or 14 hours) and incubation with shaking at 60 rpm showed no positive outcomes. Adding a washing solution to the protoplast suspension, both before and after filtration, was identified as a critical step for successful protoplast isolation. Omitting this step or using insufficient volumes led to protoplast aggregation and clumping with residual plant material, hindering separation. Among the three washing solutions tested (W5, W1, W2), only W5 demonstrated a positive effect when used at a minimum ratio of 2:3 to the enzyme solution. For purification, resuspending the protoplast sediment in a sucrose solution and overlaying it with a washing solution proved more effective, efficiently removing residual enzymatic contaminants (**Supplementary Figure S2**). However, this approach did not significantly affect yield or viability. To optimize these parameters, the following centrifugation conditions were applied: (1) 1000 rpm for 10 minutes, (2) 1200 rpm for 10 minutes, and (3) 1000 rpm for 5 minutes. It was observed that lower centrifugation forces resulted in reduced yields without improving protoplast viability.

### 3.3 Influence of the enzyme solution

The isolation of protoplasts was successfully achieved only with enzyme solutions ER1a and ER1b. The highest yields were obtained using ER1a on leaves from 1–2 weeks old *in vitro*-germinated seedlings of the ‘USO 31’ cultivar. While protoplast isolation was also achievable with ER1b using the same material, its efficiency was notably lower. Based on these results, ER1a was selected for all subsequent procedures due to its superior performance. A comprehensive overview of enzyme solution effects across donor materials is presented in **Supplementary Table S3**.

### 3.4 Influence of the plant material

**Figure 3:**
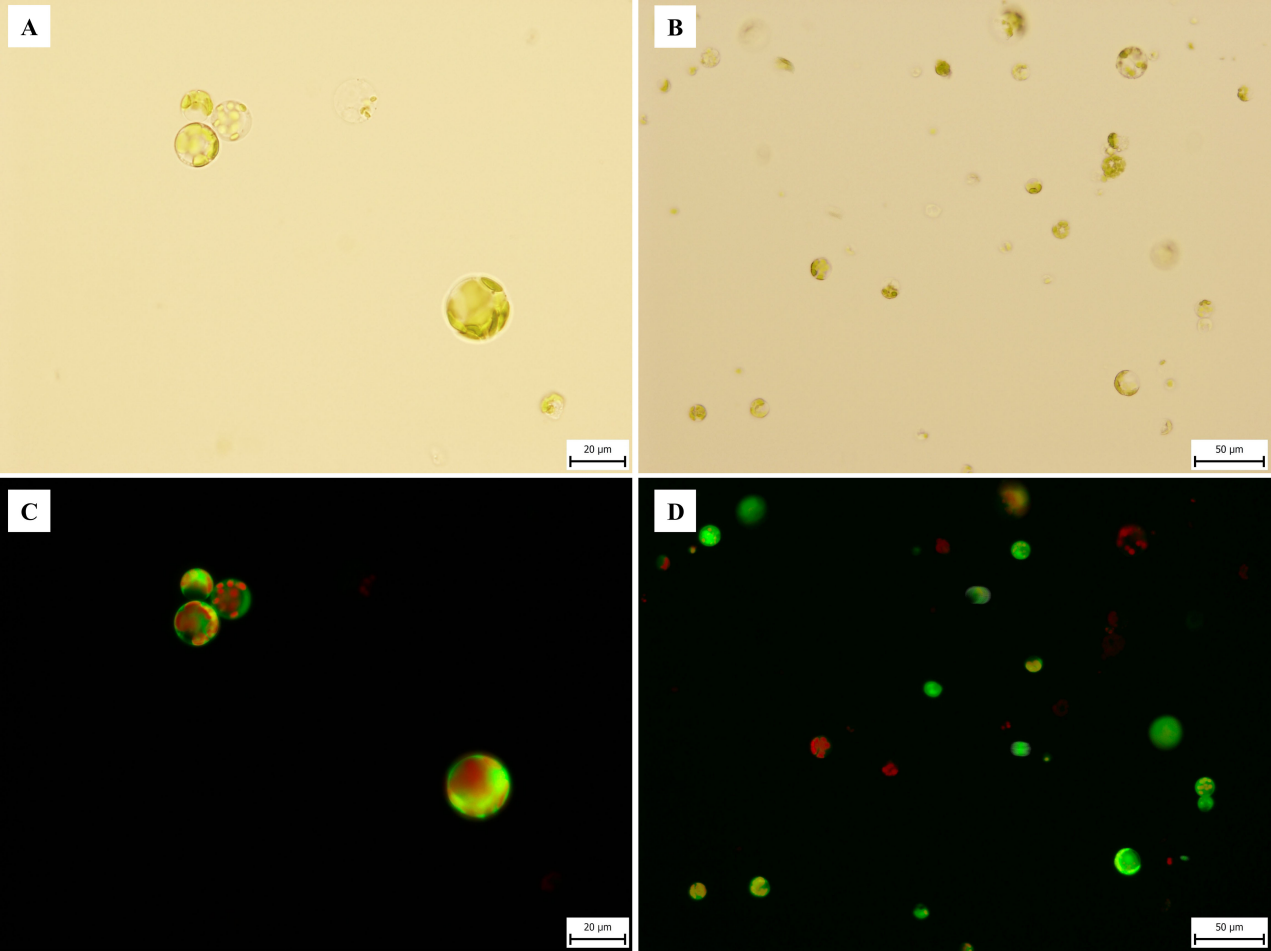
Microphotography of *C. sativa* ‘USO 31’ protoplasts isolated from 1–2-week-old leaves of *in vitro* seedlings. Freshly isolated protoplasts stained with FDA viability 83.2%. **(A, B)** Observed under transmitted light at 40× and 20× magnifications. **(C, D)** Observed under broad-spectrum UV light at 40× and 20× magnifications.

The efficacy of protoplast isolation was significantly influenced by the source and age of the plant material (**Table 4**). The best results were obtained from 1–2-week-old *in vitro*-germinated seedlings of the ‘USO 31’ cultivar. Protoplast yields in this group ranged from 4.1 · 10^6^ to 9.9 · 10^6^ cells · g^-^¹, with an average of 5.7 · 10^6^ cells · g^-^¹. The highest viability was observed at 83.2% (**Figure 3**), with an average of 66.8%. Leaves from older 3–4-week-old seedlings also yielded protoplasts, with a significantly lower average yield of 5.1 · 10^6^ cells · g^-^¹ and viability ranging between ~10–35%. For a visual comparison of the age-dependent yield, see **Supplementary Figure S3**.

**Table 4:** List of successful cannabis protoplast isolations–the impact of donor material on the yield and viability.

Leaves donor material	Yield [cells·g^-^¹]	Average yield [cells·g^-^¹]	Viability [%]	Average viability [%]
‘Finola’ 6-month-old *in vitro* cultures	4.0·10^5^	5.3 ± 1.8·10^5^	7	∼6
	6.5·10^5^		∼5	
USO 31’ 6-month-old *in vitro* cultures	7.3·10^5^	7.5 ± 0.3·10^5^	∼10	∼10
	7.7·10^5^		∼10	
USO 31’ 3–4-week-old *in vitro* seedlings	2.6·10^6^	5.1 ± 2.9·10^6^	∼10	∼18 ± 11.5
	1.7·10^6^		35	
	5.5·10^6^		25	
	7.1·10^6^		∼10	
	8.4·10^6^		∼10	
USO 31’ 1–2-week-old *in vitro* seedlings	4.2·10^6^	5.7 ± 2.4·10^6^	83.2	66.8 ± 10.5
	5.7·10^6^		70.7	
	4.8·10^6^		57.5	
	4.1·10^6^		59.2	
	9.9·10^6^		63.6	

In contrast, protoplast isolation from six-month-old *in vitro* cultures resulted in substantially reduced yields and viabilities, particularly for the ‘Finola’ cultivar (5.3 · 10^5^ cells · g^-^¹, 6% viability) and ‘USO 31’ (7.5 · 10^5^ cells · g^-^¹, 10% viability). Attempts to isolate protoplasts from plants cultivated *ex vitro* or explants transferred to *in vitro* conditions were entirely unsuccessful. Based on these findings, young leaves from 1–2-week-old *in vitro*-germinated seedlings were selected as the optimal starting material for further procedures.

### 3.5 Protoplast culture

**Figure 4:**
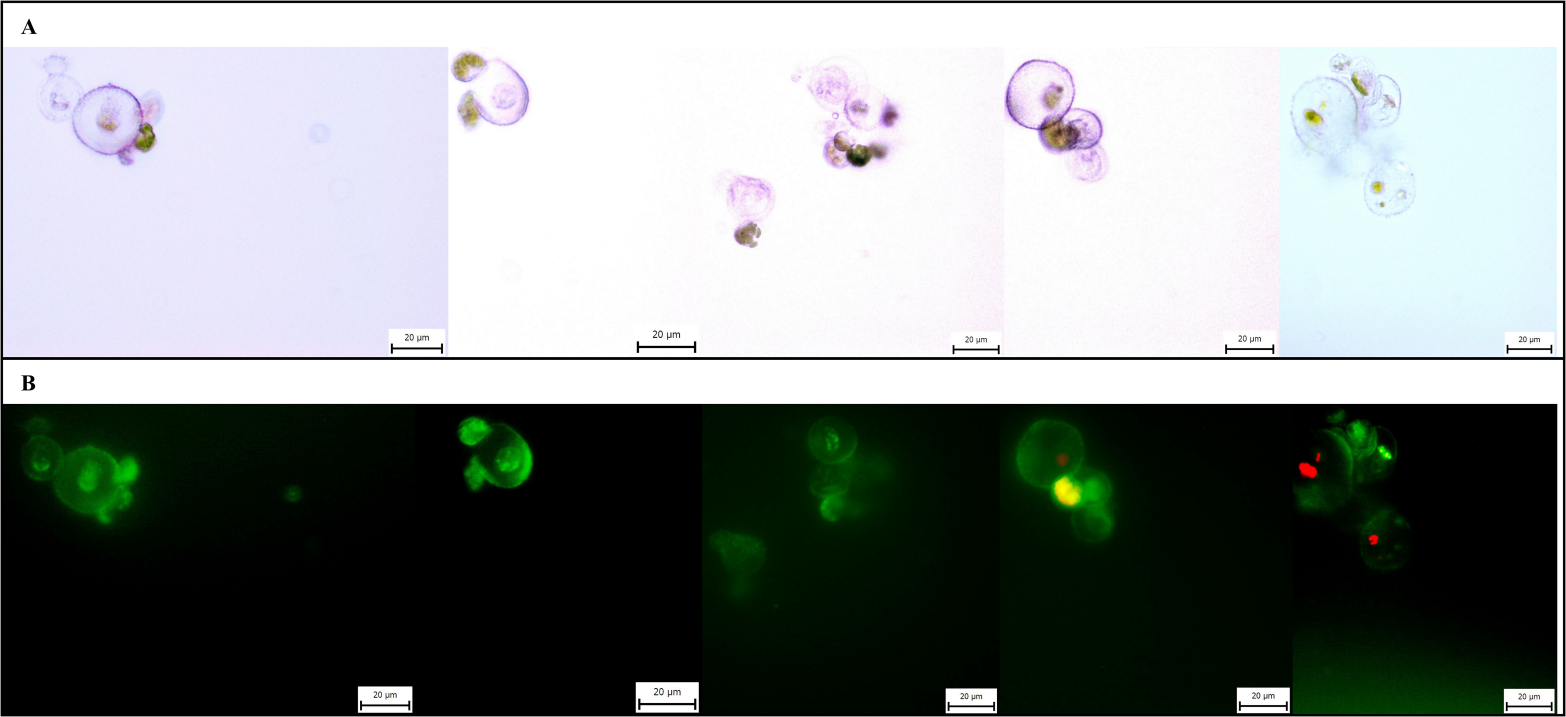
Microphotographs of microcalli of *C*. *sativa* ‘USO 31’. Cultivated for 14 days, stained with FDA, magnification 40×. **(A)** Observation under transmitted light. **(B)** Observation under broad-spectrum UV light.

Protoplast cultures were successfully established from samples with both high viability (>60%) and low viability (<15%). The results confirmed that a partially modified regeneration medium, originally developed for *Arabidopsis* thaliana, and the selected protoplast density effectively supported early proliferation in cannabis. By the end of the incubation period, the cultures remained viable, with microscopy revealing cells that had undergone at least one cell division. An extended cultivation experiment was conducted for 14 days without renewing the regeneration medium. During this period, microcalli were observed, and their cells retained viability (**Figure 4**).

### 3.6 Expression of proliferation-associated genes

**Figure 5:**
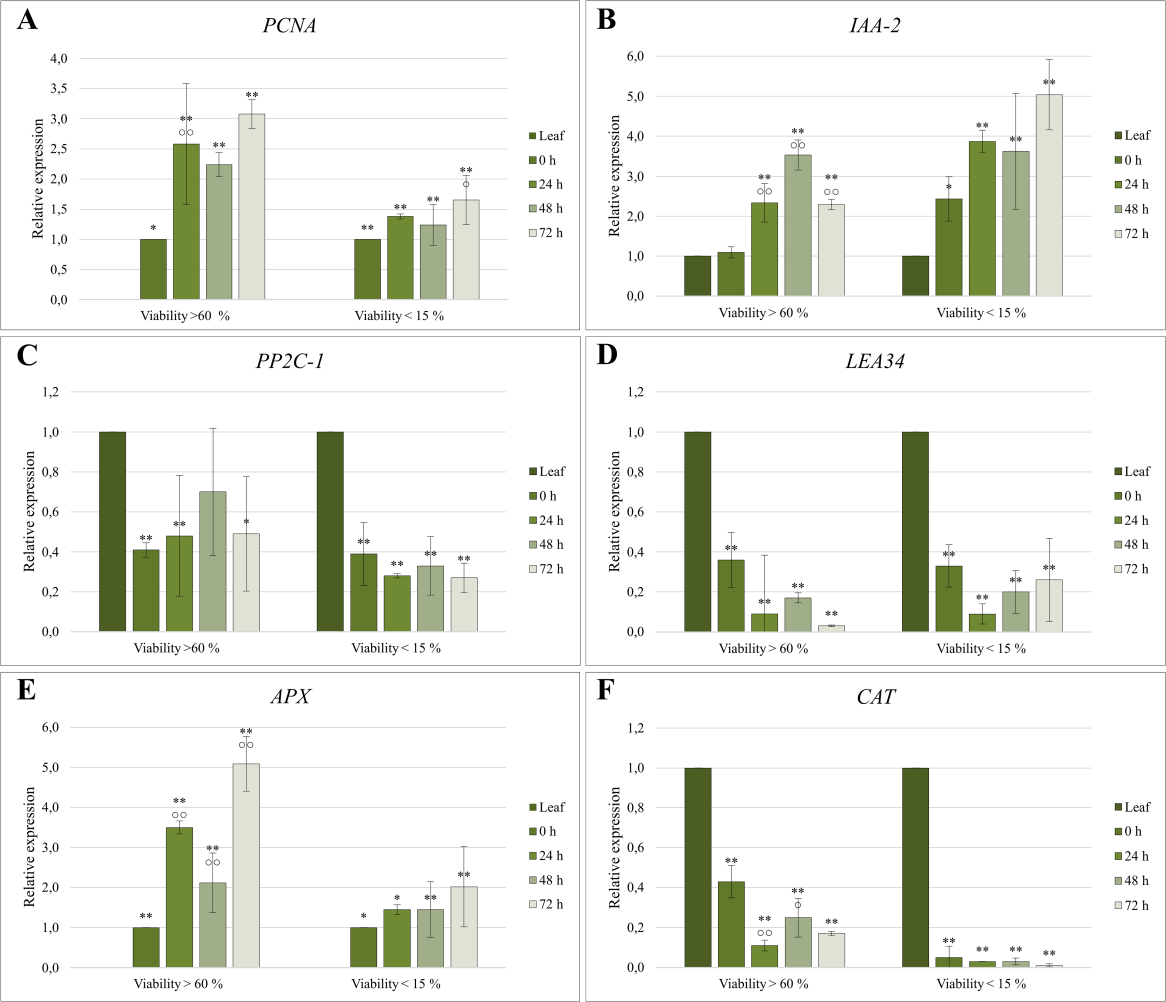
Relative expression quantification of **(A, B)** proliferation-associated, **(C, D)** abiotic stress-related, and **(E, F)** oxidative stress-related genes in protoplast cultures of *C. sativa* ‘USO 31’. Cultures with viability > 60% were derived from 1–2-week-old leaves of *in vitro* seedlings, while cultures with viability < 15% originated from 3–4-week-old leaves. Expression levels were normalized using *EF-1* as a housekeeping gene and calibrated relative to the control (Leaf) or 0 h sample. Significant differences in gene expression compared to the control are indicated by asterisks (p ≤ 0.01 “**”; p ≤ 0.05 “*”), and differences between cultivation stages are marked with circles (p ≤ 0.01 “○○”; p ≤ 0.05 “○”).

The proliferation marker *PCNA* was undetectable in the control sample (Leaf), and thus, data were normalized relative to the 0 h sample (**Figure 5**). *PCNA* expression increased significantly immediately following protoplast isolation, with the first peak observed at 24 hours. The highest expression level occurred after 72 hours of cultivation, reaching a threefold increase compared to the control. Cultures derived from protoplasts with higher viability exhibited up to twice the level of *PCNA* expression compared to those with lower viability, underscoring the relationship between protoplast viability and proliferative capacity.

The *IAA-2* gene showed statistically significant differences in expression compared to the control across nearly all samples (**Figure 5**). Both protoplast isolation and subsequent cultivation triggered an increase in *IAA-2* expression. In cultures derived from protoplasts with viability >60%, expression levels exhibited a minimal increase immediately following isolation. However, there was a notable increase during the cultivation, peaking at 48 hours with a 3.5-fold increase relative to the initial levels. By the third day, expression levels declined to those observed on the first day. Conversely, protoplasts with lower viability exhibited expression levels immediately post-isolation comparable to those of the high-viability group after 24 hours of cultivation. In this group, the highest expression was recorded at 72 hours, reaching up to a fivefold increase relative to the control.

### 3.7 Expression of abiotic stress-related genes

Protoplast isolation significantly reduced the expression of the *PP2C-1* gene, with levels declining by up to 60% compared to the donor material (**Figure 5**). Subsequent cultivation had little to no further impact on *PP2C-1* transcription.

Similarly, the expression of *LEA34* followed a comparable pattern. Protoplast isolation reduced its expression to one-third of the original level, with continued downregulation observed during cultivation. These findings highlight the substantial impact of protoplast isolation on stress-related gene expression, potentially reflecting adaptations to the enzymatic digestion process and subsequent culture conditions.

### 3.8 Expression of oxidative stress-related genes

The expression profile of the *APX* gene closely mirrored that of *PCNA*, with expression increasing over the cultivation period (**Figure 5**). The proliferation marker *APX* was not detectable in the control (Leaf). Therefore, the data were normalized relative to the 0 h sample. In protoplast cultures with higher viability, statistically significant changes in *APX* expression were observed relative to the control and across different cultivation stages. Immediately following isolation, *APX* expression increased 3.5-fold within 24 hours. During subsequent cultivation, expression initially decreased but later rebounded, reaching a maximum fivefold higher than at 0 h. In contrast, protoplast cultures with lower viability followed the same trend but exhibited smaller differences between cultivation stages, with *APX* expression levels approximately half those of the high-viability group.

In contrast, the *CAT* gene exhibited an inverse expression profile. Its expression was highest in the donor material, decreasing dramatically following isolation and cultivation (**Figure 5**). In high-viability cultures, *CAT* expression declined significantly after 24 hours, with a notable increase observed at 48 hours. In low-viability cultures, *CAT* expression was minimal and continued to decrease throughout cultivation.

## 4 Discussion

The donor material’s age, developmental stage, and cultivation conditions play a critical role in the success of protoplast isolation. Our study suggested that younger leaves are the most suitable, consistent with Evans and Bravo (2013) and Lazič (2020) findings. In contrast, attempts to isolate protoplasts from leaves of plants transferred to *in vitro* conditions, as well as from six-month-old cultures, were unsuccessful. This failure likely stems from physiological and morphological changes induced by stress during the transition to *in vitro* environments and suboptimal cultivation conditions (Evans and Bravo, 2013). Many samples exhibited senescence or vitrification, which rendered them unsuitable for protoplast isolation. These challenges may result from altered cell wall development, thinning of epidermal protective layers, or significant enlargement of vitrified cells (Pâques and Boxus, 1987; Rasco and Patena, 1997; Kemat et al., 2021). Efforts to improve protoplast isolation by shortening the enzymatic digestion period proved ineffective.

We tested various culture media, growth regulators, and container closure methods to optimize cultivation conditions. The findings confirm the beneficial effects of DKW medium, aligning with the results of Page et al. (2021). Among the tested growth regulators, 2iP yielded the most favorable outcomes, while TDZ produced the weakest response, consistent with the observations reported by Stephen et al. (2023). Reduced gas exchange under airtight conditions was identified as a critical limiting factor, as it decreases photosynthetic efficiency and forces greater reliance on nutrients from the culture medium. Elevated humidity further alters stomatal activity, increases concentrations of genotoxic H_2_O_2_, and amplifies overall cultivation stress. Under these conditions, the expression of stress and nutrition-related genes can be significantly affected, adversely impacting the cultivation process and leading to vitrification symptoms (Ševčíková et al., 2016; Xu et al., 2019; Abdalla et al., 2022; Král et al., 2022). The beneficial effects of more permeable closure observed in this study have also been demonstrated in other plant species (Majada et al., 1997; Tsay et al., 2006). Despite these optimizations, no enhancement in protoplastization efficiency was achieved.

The genetic background and genotype also significantly influence protoplast isolation outcomes, with variability observed even within plants of the same cultivar (Evans and Bravo, 2013). To ensure robust results, 9 cultivars were included in this study, representing both high and low CBD strains. Although previous studies have reported successful protoplast isolation from the ‘Finola’ cultivar (Lazič, 2020; Monthony and Jones, 2024), our findings revealed superior performance with the ‘USO 31’ cultivar. This study marks the first successful protoplast isolation from the leaves of this cultivar.

The enzymatic solution ER1a, developed by Matchett-Oates et al. (2021), proved to be the most suitable option for protoplast isolation in our study. Although the average yield was approximately 27% lower than previously reported, the maximum yield obtained in our experiments exceeded the values published by these authors. The reduced average yield may be attributed to genotypic differences between plant materials, variations in cultivation conditions, or a lower temperature used during enzymatic digestion, all known to influence protoplast isolation efficiency. Attempts to enhance isolation efficiency through the addition of pectolyase, as suggested by previous studies (Morimoto et al., 2007; Beard et al., 2021; Kim et al., 2022; Monthony and Jones, 2024), were unsuccessful. Specifically, lower pectolyase concentrations resulted in reduced yields, while higher concentrations led to complete isolation failure. These outcomes were likely caused by excessive enzymatic activity, resulting in oxidative stress and cellular damage (Ishii, 1987; 1988).

This study represents only the second report of cannabis protoplast proliferation initiation and microcallus formation, achieved earlier than previously reported (Monthony and Jones, 2024). For cultivation, we utilized a slightly modified regeneration medium originally developed for *A. thaliana* (Mathur and Koncz, 1998). The suitability of this medium for the initiation phase of cannabis protoplast cultures was demonstrated by expression analysis and successful microcallus formation.

Expression analysis of the proliferation marker *PCNA* revealed early activation, likely initiated during the isolation process, which is consistent with previous observations in other plant species (Cápal and Ondřej, 2014). This early upregulation, together with its continued increase during cultivation, supports the onset of the S-phase and indicates a strong proliferative potential of the isolated protoplasts (Williams et al., 2003; Cápal and Ondřej, 2014).; Importantly, although *PCNA* may also be expressed in non-proliferating cells (Van Diest et al., 1998), no such expression pattern was observed in our system, suggesting a close association with proliferative activity.

A significant increase in *IAA-2* expression was detected after 24 hours of cultivation, indicating heightened auxin signaling activity upon exposure to phytohormones in the regeneration medium. The up-regulation of *IAA-2* expression during successful protoplast cultivation has similarly been reported in *A. thaliana* (Pasternak et al., 2021).

*PP2C-1* and *LEA34* are associated with the abscisic acid (ABA) signaling pathway, which up-regulates their expression (Liu et al., 2019; Park et al., 2009). In our study, protoplast isolation resulted in a marked reduction in ABA signaling, which remained consistently low throughout the cultivation. Importantly, because the expression levels of these genes did not change significantly over time, it is likely that the protoplasts rapidly adapted to the *in vitro* culture conditions. These findings suggest that the cells were not exposed to substantial or increasing abiotic stress during cultivation, in contrast to the elevated stress levels observed in cannabis *in vitro* cultures (Král et al., 2022).

Oxidative stress, a critical factor affecting protoplast viability, was assessed through the expression of two key antioxidant genes, *APX* and *CAT*. These enzymes act within interconnected pathways linked by a shared substrate and display a dynamic balance during stress response (Caverzan et al., 2012; Ratanasanobon and Seaton, 2013). In our system, *APX* showed dominant upregulation during early cultivation, suggesting the primary role of the ascorbate–glutathione cycle in ROS detoxification. In contrast, *CAT* played a complementary role. Elevated expression levels of both genes correlated with reduced ROS levels, which is essential for chromatin reorganization and the initiation of dedifferentiation (Ondřej et al., 2008; 2010). The consistent expression trends of *APX* and *PCNA* genes further underscore the coordinated mechanisms driving protoplast proliferation. The functional relevance of *PCNA*, *APX*, and *CAT* expression was further supported by comparisons between protoplast cultures of differing viability. Cultures with high viability (>60%) maintained robust expression of all three genes, reflecting a strong capacity for proliferation and oxidative stress mitigation. In contrast, low-viability cultures (<15%) showed markedly reduced expression all three genes, consistent with impaired stress responses and limited proliferation potential (Ondřej et al., 2010).

## 5 Conclusion

This study successfully established a protocol for the *Cannabis sativa* L. protoplast isolation and cultivation using young leaves from *in vitro*-grown ‘USO 31’ seedlings. The application of a partially modified regeneration medium, originally designed for *Arabidopsis thaliana*, enabled the initiation of cannabis protoplast cultures. Transcriptomic analyses revealed that the protoplast cultures were viable and exhibited robust antioxidant responses, with stress levels lower than those of *in vitro*-germinated plants. Importantly, the protoplasts progressed into the S phase of the cell cycle, underscoring their potential for further developmental studies. By reporting only the second successful cultivation of cannabis protoplasts, this work lays the foundation for future research into cannabis protoplast biology and applications. The insights gained here could significantly advance research and development in the largely unexplored domain of cannabis protoplast cultures.

## Data Availability

The datasets presented in this study can be found in online repositories. The names of the repository/repositories and accession number(s) can be found in the article/**Supplementary Material**.
