# Supplementary material for: Early protoplast culture and partial regeneration in Cannabis sativa: gene expression dynamics of proliferation and stress response
Source: Front Plant Sci. 2025 Jun 6;16:1609413. doi: 10.3389/fpls.2025.1609413 (PMC12179117; doi:10.3389/fpls.2025.1609413)
Supplement: Supplementary file 1 [file DataSheet1.docx]

**Fig. S1**

**
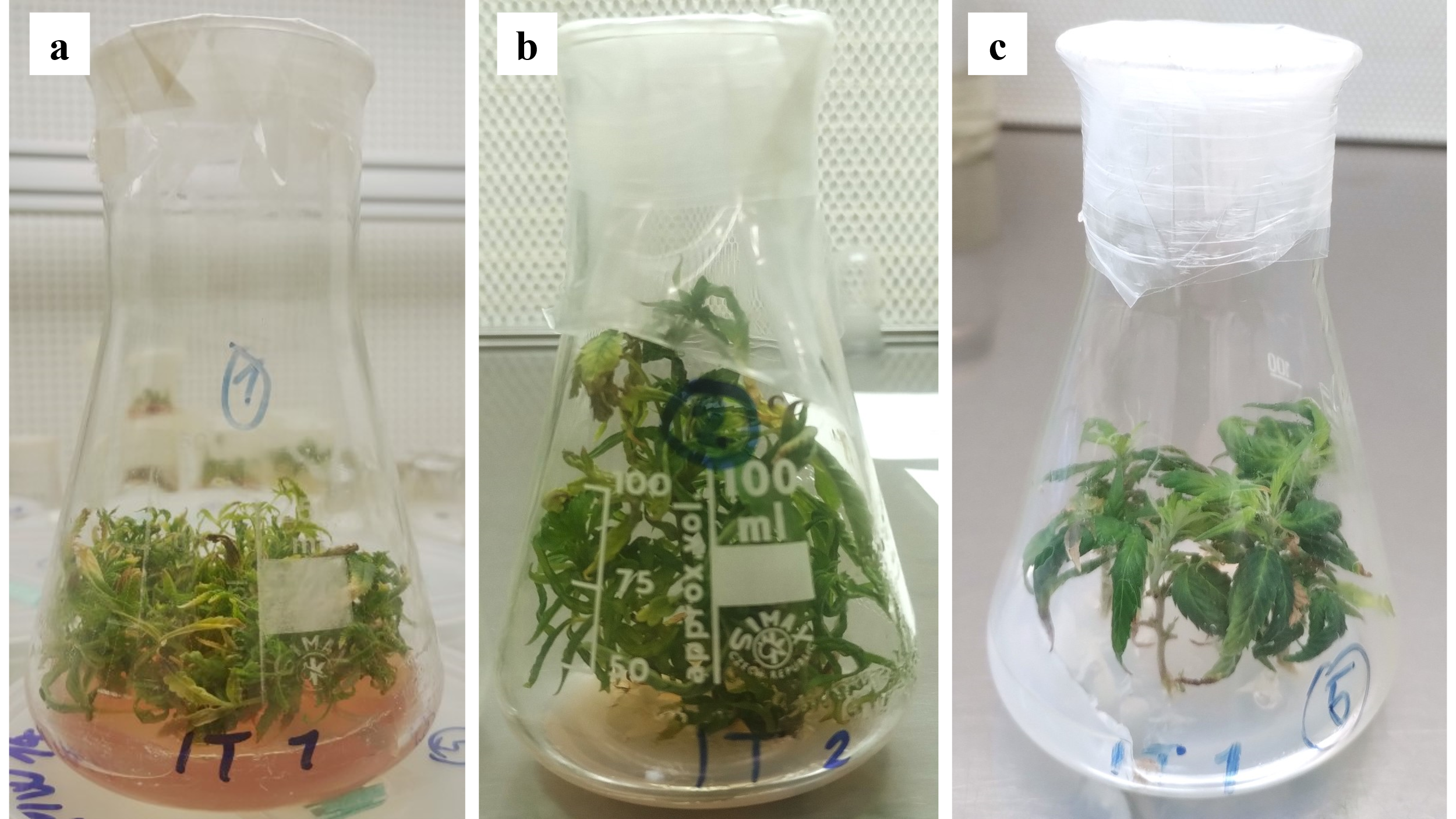
**

***C. sativa in vitro* – Effect of culture media**. Four-week-old cultures of 'Eletta Campana' derived from nodal explants. (**a**) Cultured on medium KM1. (**b**) Cultured on medium KM2. (**c**) Cultured on medium KM5.

**Fig. S2**

**
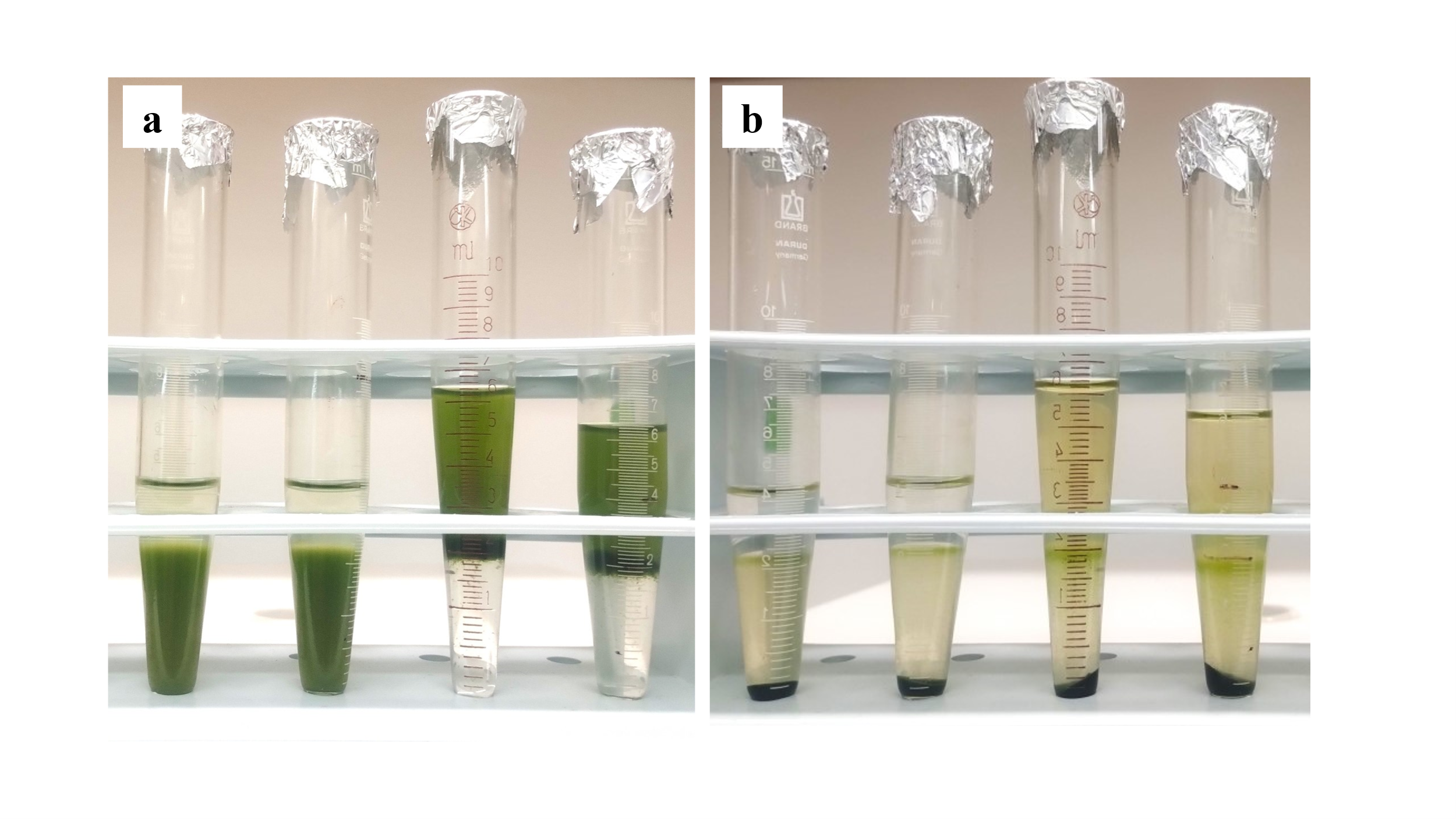
**

**Comparison of Protoplast Purification Efficiency.** (**a**) Protoplast suspensions before centrifugation in a sucrose gradient. (**b**) Different levels of purification observed after centrifugation, with brown discoloration in the solution above the floating protoplasts.

**Fig. S3**

**
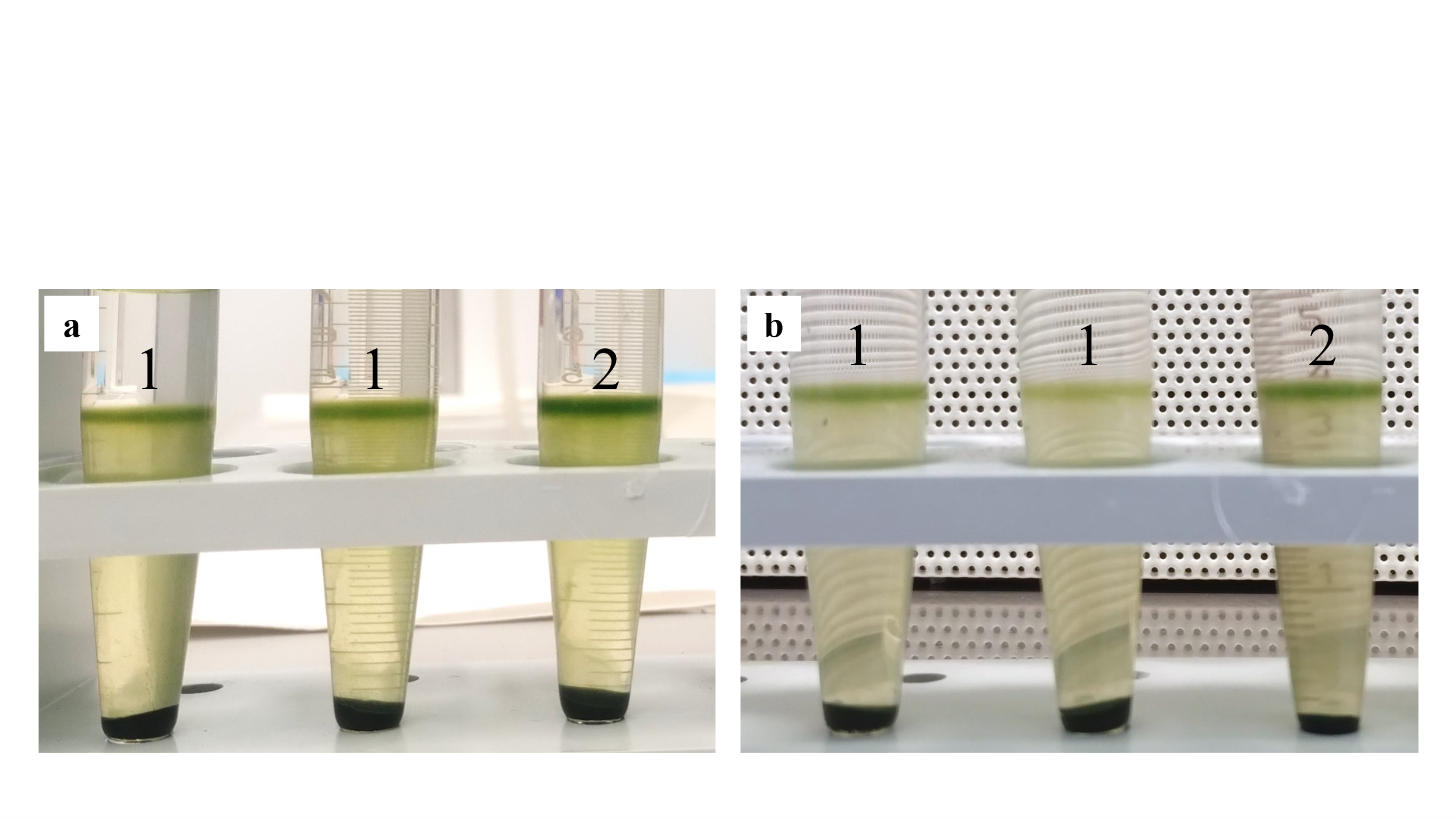
**

Visual comparison of protoplast yield. (**1**) Protoplast samples isolated from leaves of 3–4-week-old in vitro USO 31 seedlings. (**2**) Protoplast samples isolated from 1–2-week-old seedlings. (**a**) Experiment 1. (**b**) Experiment 2.
